# Biocompatible Polymer Materials with Antimicrobial Properties for Preparation of Stents

**DOI:** 10.3390/nano9111548

**Published:** 2019-10-31

**Authors:** Kateřina Škrlová, Kateřina Malachová, Alexandra Muñoz-Bonilla, Dagmar Měřinská, Zuzana Rybková, Marta Fernández-García, Daniela Plachá

**Affiliations:** 1 Nanotechnology Centre, VŠB–Technical University of Ostrava, 17. listopadu 15, 708 00 Ostrava-Poruba, Czech Republic; katerina.skrlova@vsb.cz; 2 Center of Advanced Innovation Technologies, VŠB–Technical University of Ostrava, 17. listopadu 15, 708 00 Ostrava-Poruba, Czech Republic; 3 Department of Biology and Ecology, Faculty of Science, University of Ostrava, Chittussiho 10, 71000 Ostrava, Czech Republic; katerina.malachova@osu.cz (K.M.); zuzana.rybkova@osu.cz (Z.R.); 4 Institute of Polymer Science and Technology (ICTP-CSIC), Juan de la Cierva 3, 28001 Madrid, Spain; sbonilla@ictp.csic.es (A.M.-B.); martafg@ictp.csic.es (M.F.-G.); 5 Faculty of Technology, Tomas Bata University in Zlín, Vavrečkova 275, 760 01 Zlín, Czech Republic; merinska@utb.cz; 6 ENET Centre, VŠB–Technical University of Ostrava, 17. listopadu 15, 708 00 Ostrava -Poruba, Czech Republic

**Keywords:** stent, biodegradable polymer, polylactide, antimicrobial agents, antimicrobial effects, medicine

## Abstract

Biodegradable polymers are promising materials for use in medical applications such as stents. Their properties are comparable to commercially available resistant metal and polymeric stents, which have several major problems, such as stent migration and stent clogging due to microbial biofilm. Consequently, conventional stents have to be removed operatively from the patient’s body, which presents a number of complications and can also endanger the patient’s life. Biodegradable stents disintegrate into basic substances that decompose in the human body, and no surgery is required. This review focuses on the specific use of stents in the human body, the problems of microbial biofilm, and possibilities of preventing microbial growth by modifying polymers with antimicrobial agents.

## 1. Introduction

The human body has a complex structure, where each organ has an important task. Body organs are composed of various cell types, extracellular matrices, proteins, and other macromolecules. The organs are supplied with body fluids that are transported by passive diffusion processes or tubular organs. These tubular organs differ in the structure of epithelial cells being adapted to the liquid or solid contents that flow through the tube, such as blood, bile, food, fluids, etc. [1,2]. Diseases of blood vessels and other body ducts are a considerable problem of contemporary medicine. Severe illnesses cause their narrowing and clogging and the patients suffer from severe pain [3]. One of the possible solutions to this problem is the use of stents. Stents are small tubes made of metal or polymer usually used for restoring the passage of blood vessels or ducts. They also have a wide range of uses in other parts of the body. According to their location in the body, they can be divided into coronary and vascular, esophageal, duodenal and other intestinal ducts, biliary, pancreatic, ureteral and prostate, or drug stents [4,5,6,7,8,9,10].

One of the main complications in the use of stents is the formation of microbial biofilm on their inner surface. In particular, bacteria irreversibly adhere to the surface of medical objects, creating a biofilm that causes severe infection. The biofilm resists immune protection system and antibiotics therapies, and it can cause serious illness or even lead to the death of patients. There are several possibilities for how to prevent a bacterial adhesion and growth on the surface of medical devices. One of them is to prepare a material on which bacteria and other microorganisms are not able to adhere and develop into the biofilm. Currently, materials based on polymer composites or polymers with surfaces modified with an antimicrobial agent are studied for their efficiencies to prevent biofilm growth. In addition, other options based on physical surface properties such as surface roughening, or vice versa smooth surface, super hydrophobic, etc. were designed to prevent bacterial adhesion. This review is focused on the research and development in medical stents, especially biliary stents, and on the possibilities of the use of new materials for stent production to increase their ability to resist microbial contamination and increase the quality of their utility properties. It consists of three parts: (i) the first part focuses on the overview of stents used in medical applications and also on problems arising from biofilm formation, (ii) the second part is focused on biliary stents and (iii) in the third part new possibilities for stent production preventing microbial growth are discussed.

## 2. Stents

### 2.1. The Principle of Stents

**Figure 1:**
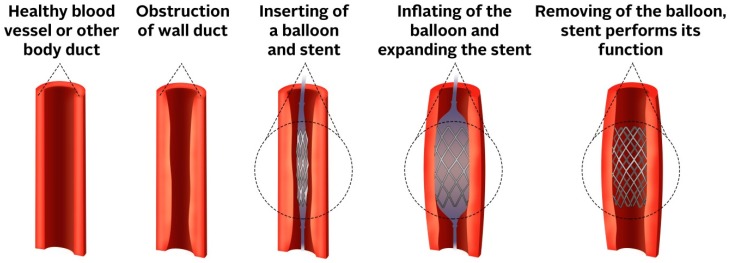
The principle of inserted stent.

Stents are used for restoring the patency of a blood vessel and other ducts in the body. When an obstruction is created in a duct, blood or other body liquids cannot flow properly. In that case, an endoscopically inserted stent is used. When an expandable stent is used, it is often inserted with a balloon that is inflated after the stent has been correctly positioned, and then the stent is stretched to the desired width. A schematic representation can be seen in Figure 1.

### 2.2. Types of Stents

**Figure 2:**
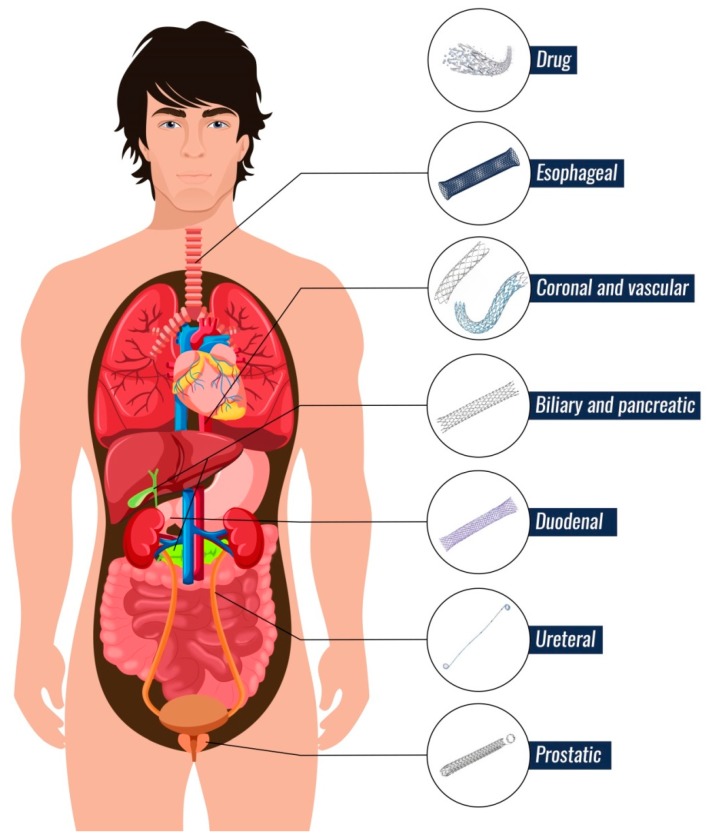
Nine main types of stents.

There are nine major types of stents that are divided by location in the human body (Figure 2): Coronary and vascular, esophageal, duodenal, biliary, pancreatic, ureteral, prostate and drug stents. Stents can be made of three materials: (i) metals, (ii) permanent polymeric materials and iii) biodegradable polymeric materials. The type of material is selected according to the location of the stent in the human body and according to the duration of its action. The material choice also depends on the cost of the stent. Currently, efforts are being made to replace metal stents by biodegradable polymer stents. The disadvantages of metal stents are their easy clogging, stent migration in the human body, cell ingrowth into the stent and restoration of stenosis, problems during some special medical examinations such as MRI (Magnetic Resonance Imaging) and the high price of stent. These problems may also occur in case of use of permanent polymer stents. The main complication of stents made from permanent materials is a surgical intervention to remove the stent as surgery can endanger a patient’s life [11,12,13,14]. For this reason, new possibilities of manufacturing and modifying materials are being studied to reduce the risk to the patient. Biodegradable stents disintegrate into essential components that the human body can safely break down and eliminate. In addition, various antibacterial agents are used to modify polymeric materials to reduce the risk of infection and biofilm formation on the stent surface and consequently stent clogging [12].

#### 2.2.1. Vascular and Coronary Arteries

Cardiovascular diseases are the most prevalent diseases in the world. In 2015, 40% of all mortality was caused by a cardiovascular and circulatory system disease [15,16]. The main cause of these diseases is the deposition of fat and cholesterol in the coronary artery walls. Fat and cholesterol can block the coronary vessels. This problem is solved by angioplasty, during which a balloon catheter is used which inflates at the stenosis site. The balloon compresses the plaque on the walls of the coronary arteries, and after its removal ensures the continuous flow of blood. The role of the stent in that case is to maintain blood flow [17]. The first coronary stent was made in the late 1980s. Since then, coronary stents have developed very rapidly. They are classified into three main groups: Bare metal stents (BMS), drug-eluting stents (DES) and bioresorbable vascular scaffolds (BRS) [6,18,19,20,21].

Bare metal stents (BMS) have been produced since 1986, and were first manufactured from stainless steel. Currently, cobalt-chromium, platinum-chromium or other alloys have largely replaced stainless steel. An ideal BMS should have good deliverability, flexibility, low thrombogenicity and good biocompatibility [18].

Drug-eluting stents (DES) are relatively new in medicine. The first models (first phase models) were applied in 1999. They had a more complex structure as they gradually released a drug. They consist of a metal part and a polymeric cover which contained a drug (Myolimus, Novolismus and other antiproliferative drug). However, the polymeric coating is one of the causes of the pathogenesis of long-term stent failure by inducing a potential chronic inflammation [18].

The further models (second phase models) use biodegradable polymers or bioresorbable materials in combination with metal stents. The most widespread biodegradable polymers for drug-eluting stents are poly-L-lactic acid (PLLA) and its copolymers (such as poly-DL-lactide-*co*-glycolide). The polymer contains a drug, which is gradually released. After degradation of the biodegradable polymer in its early phase, the metallic platform of the stent remains in the coronary artery. The polymer degrades to low molecular weight substances that are excreted from the body. The polymers used must be well characterized because when mixed with another polymer, the molecular weight, structure, and other physical and chemical conditions affect the polymer degradation time and its biocompatibility [22,23]. The first clinically available polylactide stent was manufactured by the company Abbott in 2011 and was approved by the FDA in July 2016. It was made from PLLA and was called "ABSORB 1.0". ABSORB 1.0 was described as a temporary scaffold for the treatment of ischemic heart disease. The first generation of ABSORB 1.0 had a zigzag structure with a cross profile of 1.4 mm and strut thickness of 150 µm [24]. 

The second generation of ABSORB 1.0 was called "ABSORB 1.1". The stent had many shared features with the first generation. However, the stent manufacturing process was different. The polymer was blow into a mold to provide it with prolonged radial support [25]. The company Abbot is trying to develop a thinner reinforcing stent, which will be named "Falcon" and its strut diameter will be less than 100 µm [26,27].

In 2013, another company, Elixir Medical, developed their first stent called "DESolve 150". This stent was bioresorbable polylactic acid (PLA) based and it had strut thickness of 150 µm incorporating 2 platinum-iridium markers at both ends [28,29]. The main difference between DESolve and ABSORB was its ability of intrinsic radial expansion, which also provides a greater tolerance to overexpansion for DESolve. The skeleton of DESolve was coated by PLA, and this PLA coating was impregnated with Myolimus, which is an antiproliferative drug that inhibits the growth of a cell. This stent is resorbing within 12–24 months through hydrolysis [30,31]. The next steps of Elixir Medical were in the development of stents with lesser strut profile. In 2014, they introduced the DESolve 100, a stent with a strut profile of 100 µm, and degradation time within one year. It is fully a biodegradable PLLA-based stent that elutes Novolismus with anti-proliferative and anti-inflammatory properties [28,32]. Currently, they are working on new DESolve Cx stents with an average strut thickness of 120 µm and DESolve NTx stents which are third-generation vascular stents [33].

Meril Life Sciences (City, India) has tested the efficacy of its biodegradable stent in an experimental animal study [34,35]. The stents were made from PLA, and its strut thickness was smaller than 200 µm. The stent released Sirolimus over 90 days with a dosage 1.25 g/mm^2^. Three platinum markers were inserted into the stent for a better tracking of the stent placement in the body [36]. 

Vascular and coronary stent insertion is accompanying with the risk of microbial biofilm formation, which can cause serious infectious diseases. Another risk of coronary stents is cell ingrowth into the stent and restoration of stenosis [37,38].

#### 2.2.2. Esophageal Ducts

Malignant and benign esophageal diseases are a serious problem in which the esophagus narrows. Food and fluid are difficult to get into the stomach, and the patient suffers from severe pain. The main disease that affects the esophagus is cancer [39]. This cancer causes a rapid death; only less than 20% of patients with this disease will survive more than five years. Esophageal stents are a good solution for patients because this palliative treatment allows per-oral drinking and feeding, as well as the swallowing of saliva [40]. For the treatment of malignant and benign esophageal diseases, self-expanding polymer stents (SEPS) and self-expanding metal stents (SEMS) are used. SEPS have several advantages over SEMS. They are cheap, their placement is easy, and they do not induce a tissue reaction [8,41,42]. However, polymeric stents have a high migration rate to the remaining digestive tract [43].

For that reason, the SEMS are currently used for the treatment of malignant esophageal obstruction. They have a mesh structure that helps to self-expand after stent insertion in the esophagus, and, consequently, it restores passage of the esophagus. The use of SEMS is frequent, but these stents have some complications, which are caused only by the mesh structure of the stent. Such a structure does not prevent the new tissue from growing through the mesh, which then grows through the stent and can bleed [43]. This leads to a risk to a patient’s life and the stent must be surgically removed.

Biodegradable polymeric stents can be one of the solutions to this problem. They are made of polylactide or polydioxanone [44,45,46]. These types of stents will also be useful for patients with temporary esophageal injuries or esophageal cancer patients, since the stent disintegration time may be affected by the stent chemical composition. Biodegradable polymeric stents need not be removed even after migration to another part of the digestive system, and have shown good results in the treatment of benign esophageal strictures [47].

#### 2.2.3. Duodenal and Pancreatic Ducts

Pancreatic cancer is one of the diseases that usually leads to the death of a patient. This cancer can be treated by surgical resection at an early stage, but more than 80% of patients are diagnosed at an unresectable stage [48]. Carcinoma of pancreatic, stomach, duodenum, or proximal jejunum can cause gastric outlet obstruction (GOO). This complication occurs in 10–20% of cases of pancreatic-biliary cancer [10,49]. GOO is treated either by a surgical gastrojejunostomy or by endoscopic duodenal stenting, which was first reported in the early 1990s. The latter method is a non-invasive treatment option for GOO patients. Both methods were compared, and studies revealed no differences in the incidence of adverse events and the overall survival between them. Endoscopic stenting has some advantages: A shorter hospital stay, and less strain on the body than gastrojejunostomy. Some studies indicate that a stent with chemotherapy treatment can extend a patient’s life by up to three months [10,50,51,52]. Another gastrointestinal area where stents are used is the small bowel, or they can be applied for the treatment of colorectal cancer due to intestinal strictures, which are complications of enteral diseases [53,54].

Currently, the treatments of pancreatic diseases are continuously evolving. Pathological conditions are treated using endoscopic therapy. One method of endoscopic therapy is an insertion of a pancreatic stent. Pancreatic stents are important for the treatment of recurrent idiopathic pancreatitis, chronic pancreatitis, pseudocysts, pancreatic fistulae and main pancreatic duct injuries, complications of acute pancreatitis, and in the prevention of post-endoscopic retrograde cholangio-pancreatography. Pancreatic stents are usually used to treat obstructing stones using bypass or to treat intraductal hypertension and for restoring lumen patency in cases where major pancreatic duct has been disrupted, in dominant symptomatic constrictions, in fluid collections or drainage pseudocysts, to treat symptomatic major or minor papilla sphincter stenosis, and to prevent procedure-induced acute pancreatitis [9]. Pancreatic stents vary greatly depending on the type of treatment. Stents differ in shape and material from which they are made. Currently, self-expanding metal stents are the most commonly applied; however, they have several disadvantages including the stent occlusion together with inward or outward migration and anatomic changes of the pancreatic duct. These problems limit their long term use [55].

#### 2.2.4. Biliary Ducts

One of the current problems of gastro medicine is the benign stenosis of biliary tracts. The benign stenosis is manifested by a narrowing of the bile ducts or even biliary obstruction. Strictures are the result of injuries to the bile ducts, gallstones, chronic inflammatory disease, gallbladder surgery, or gallbladder cancer [56,57,58,59,60]. Biliary obstruction may also occur as a result of pancreatic cancer. It is a very serious illness that is manifested by jaundice and itching, but occasionally may progress to biliary sepsis secondary to cholangitis and also causes severe pain to patients. The solution to this problem is the use of stents that pass through the bile duct; however, a formation of biofilm in combination with a high density of biliary fluids is a common complication when they are used. This problem is the subject of this review, and a separate chapter is devoted to biliary stents [61,62].

#### 2.2.5. Ureteral Ducts

First generation ureteral stents were made from silicone, which was replaced by polyethylene, but polyethylene becomes unstable in urine and leads to fractures. Gradually, stents have been improved and currently two-layer stents from polyurethane are being used. Ureter stents are used to prevent ureteral obstruction, to facilitate the passage of stone fragment, and to prevent delayed ureteral stricture formation [63]. Ureteric stents are used primarily for pain relief at an obstruction. Ureteric stents have some disadvantages such as infection, pain drainage failure, dislodgment, frequent urge to urinate, and migration. These problems can cause damage to the kidneys or their failure. There is not currently an ideal ureteric stent [64] since they are very favorable for the adhesion of microorganisms and subsequent biofilm formation. Biofilm clogs the stent and this leads to widespread infection and sepsis. It is very resistant to antimicrobial agents [65].

#### 2.2.6. Prostatic Ducts

Benign prostate hyperplasia (BPH) is a benign prostate enlargement associated with aging that can lead to urinary disorders. This problem is being treated with a transurethral resection of the prostate (TURP). This treatment involves surgery, which is often risky in older patients and therefore permanent catheterization is the only alternative. The prostatic stents are an alternative to conventional surgical therapies and restore urine flow by maintaining the prostate lumen [66]. Prostate stents can be divided into temporary and permanent. Temporary prostatic stents keep the lumen of the urethra open and are not incorporated into the urethra wall. Non-absorbable and biodegradable materials are used to produce prostate stents. Stents are made of stainless steel, nitinol, polyurethane, polyglycolic, or polylactic acid. Non-absorbable stents must be withdrawn between 6–36 months. The first generation of prostatic stents were made from stainless steel and were called Urispiral and ProstaKath [67]. The incidence of complications in these stents was significant. The most common complications being stent migration, recurrent urinary infection, and hematuria with urinary retention due to the formation of clots and incrustations [68]. The development of modern materials brought stents of second generation that are made from nitinol and polymeric material such as polyurethane, polylactide, and copolymers of polylactide with polyglycolic [69]. Permanent stents were initially developed for vascular and coronary applications and were later adapted for urology. The urethral wall grows through the stent. This process reduces the risk of urinary infections and migration but has the disadvantage of a difficult removal, if necessary [70]. Prostatic stents are susceptible to biofilm formation as ureteral stents.

## 3. Biofilms

**Figure 3:**
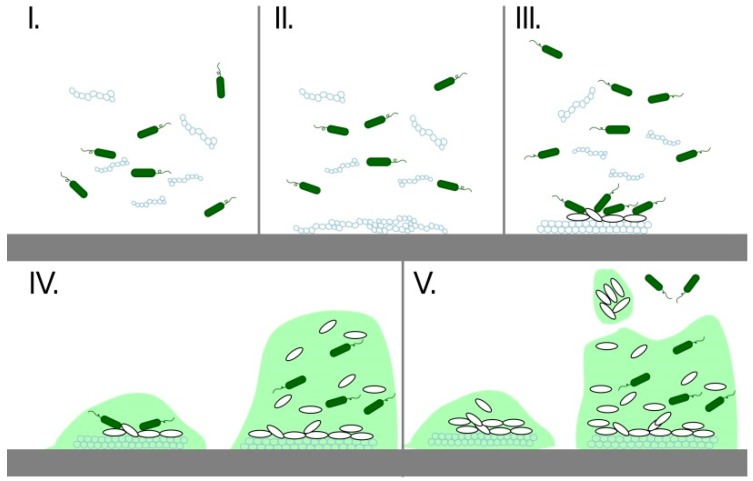
Five steps of biofilm formation: I. Host products (**blue chains**) are attached to the biomaterial, II. Creation of organic film, III. Attachment of microorganisms (**green lozenges**), creating of extracellular polymeric matrix (**white lozenges**), IV. Development of the biofilm, V. The release of cells from the biofilm.

As mentioned above, the most common problems of stents are due to their clogging due to the formation of biofilm and adhesion of biological substances that pass through the stent. Biofilm represents the most widespread way of microbial growth in nature and is critical for the development of clinical infections. It is produced by bacteria and host products [71,72] that tend to adhere to different surfaces. They create huge colonies known as biofilms. Existence in biofilms is for them more advantageous because biofilm provides protection for their cells, maintains a homeostasis, and creates a barrier that isolates bacteria from the surroundings. There is extensive communication between bacteria and the effective horizontal transfer of resistance and virulence genes that make bacteria very resistant. Thus, bacteria cells in biofilm are more resistant to toxic substances, UV radiation, mechanical damage, bacteriophages, or predators. They are also more resistant to the immune system and antibiotics in a human or animal body [73]. The surface of the material is very quickly occupied by bacteria because the host products create a biofilm on the material that helps to bind the bacteria. This process takes place in five stages, as can be seen in Figure 3 [74].

Biofilms affect many medical applications and are a serious medical problem at the present time. They are found, for example, in catheters, stents, contact lenses, and many other implants. Biomaterials at risk of attaching bacteria together with corresponding body fluids and major components of the host products for bacterial growth are classified in Table 1 [75]. The biofilm’s high resistance to antibacterial agents results in a great risk of infection and therefore creates a very hazardous situation for patient health [65,76]. Microbial cells that live in biofilms have greater resistance to antimicrobial substances than cells that live alone in planktonic form [73,77]. Due to this resistance, the treatment of biofilm infections is very difficult. Infected implants must be removed from the patient’s body. This procedure is usually expensive and dangerous for patients because surgical procedures can cause other complications [73,78,79]. Millions of implants and catheters are used for treatment every year. Table 2 shows examples of implants prone to the infections of biofilms. 

For this reason, new materials are being developed to reduce the risk of biofilm formation and infection. These materials are based on polymer composites or polymers with surfaces modified with an antimicrobial agent. In addition, other options based on physical surface properties such as surface roughening, or vice versa smooth surface, super hydrophobic, etc. should be considered. 

## 4. Biliary Stent

As was previously stated, biliary stents are used for the treatment of biliary obstructions. They are very vulnerable to the formation of biofilm due to the environment in which they are used and also to the nature of the fluid flowing through them. Thus, new antimicrobial materials are challenged to solve the problem of biliary obstructions.

The usage of biliary stents is non-invasive and brings pain relief to patients. The procedure is often used for the treatment of advanced irremovable tumors [86,87]. Biliary obstruction may be caused by intrahepatic and extrahepatic causes. Intrahepatic causes are most often hepatitis, cirrhosis, or the excessive use of drugs. Hepatitis is the inflammation of the liver, which is caused by viruses, drugs, and alcohol. Cirrhosis is a disease that damages and disorganizes the internal structure of the liver, and consequently it causes chronic inflammation. Drugs and anabolic steroids may cause cholestasis and can increase the risk of developing gallstones, which are the most common cause of a biliary obstruction. Extrahepatic causes include stone disease, cholangitis, biliary stricture, neoplasms, and parasites. Neoplasms are tumors that cause a biliary obstruction. Cholangitis can cause carcinomas of the biliary epithelium, gallbladder carcinomas, and carcinoma of the ampulla of Vater. Every carcinoma causes a tapering or clogging of the biliary tracts. The standard treatment is surgery [88,89,90], but only about 20% of these patients are resectable [91,92]. Patients who have unresectable tumors have a poor prognosis of quality of life and survival [60,91,93]. Another solution for the treatment of stenosis is the endoscopic insertion of a biliary endoprosthesis—a stent. This solution is non-invasive and also allows other treatment possibilities such as radiation therapy, chemotherapy, etc. This method is usually palliative, but endoscopic drainage can support the patient’s feeling of well-being [94]. Biliary stents are produced from a polymer or metal materials [95]. They are used to restore biliary patency, they facilitate the drainage of bile into the digestive tract, and they are applied in the palliation of malignant biliary obstruction as well as in benign conditions such as benign biliary strictures or biliary fistulas.

Stents are classified into two types: (i) uncovered and (ii) covered. The covered stents are coated with a synthetic or biological membrane, which prevents cell ingrowth into the stent. However, the covered stents are associated with little flexibility and the risk of migration in the digestive tract [96]. A suitable stent for the biliary tract should have a large diameter, great flexibility, high expansion ratio, easy delivery system, the cell should not have ingrowth into the stent, and should not need repeat surgery [96,97].

Since their introduction in 1979, endoscopic biliary stents have become the first choice method to treat cholestasis for a malignant or benign biliary obstruction [95]. The success rate of this method exceeds 90% and complications of this procedure are rare. Currently, self-expanding metal stents are used. The wider diameter allows perfect drainage, thus reducing probability of stent clogging. The disadvantages of metal endoprosthesis include its susceptibility to tumor ingrowth, impossibility of removal, and its high cost. For these reasons, polymer stents are studied [98].

### 4.1. Biliary Obstruction

**Figure 4:**
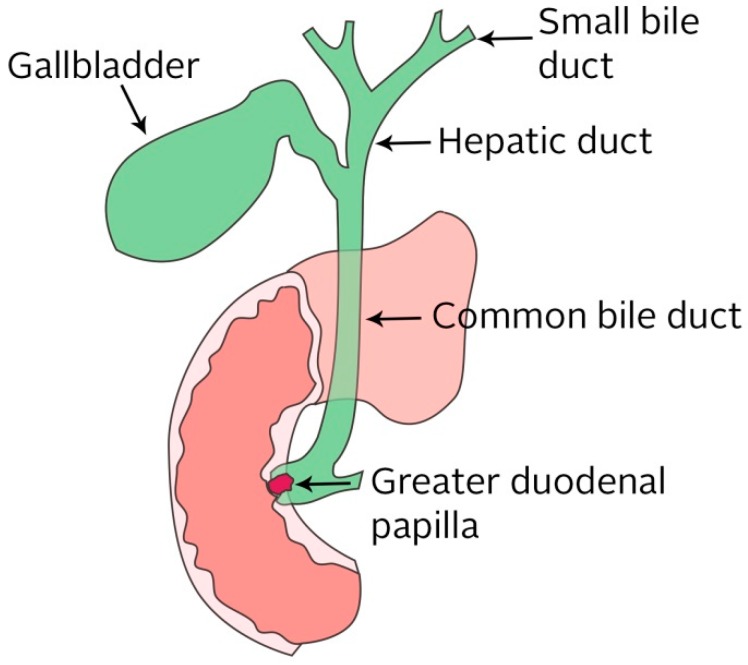
Diagram of biliary tracts.

Biliary obstruction is a disease of the biliary tracts. The biliary system in the body is a system of small ducts that connects various organs (Figure 4). It affects a significant portion of the worldwide population. This disorder may be due to biliary stones, gallbladder surgery, chronic inflammatory disease, or more serious diseases such as pancreatic cancer, cancer of the biliary duct, or external compression secondary to lymph node metastasis [99].

**Figure 5:**
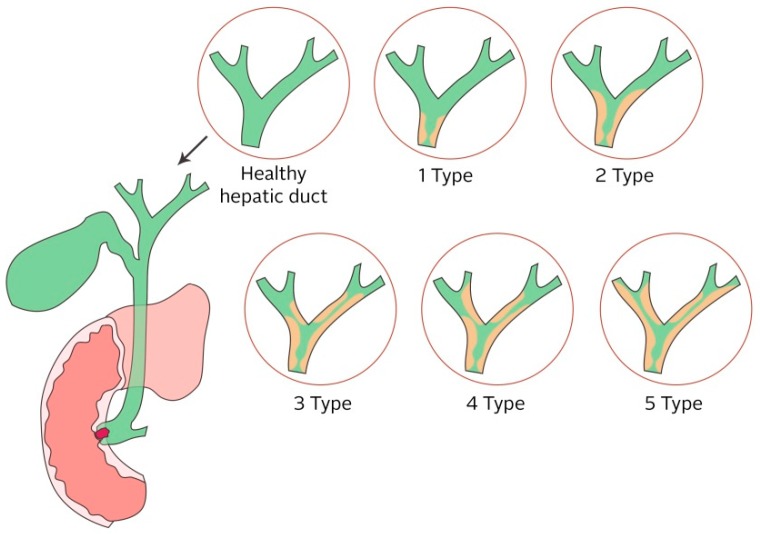
Types of biliary obstruction classified by Bismuth [105]: 1 Type: An obstruction is located further than or equal to 2 cm from the main hepatic confluence, 2 Type: An obstruction is located less than 2 cm from the main hepatic confluence, 3 Type: The ceiling of the biliary confluence is intact, right ductal system communicates, 4 Type: The ceiling of the biliary confluence is intact, left ductal system communicates and 5 Type: The ceiling of the confluence is destroyed, bile ducts are separated.

The bile ducts lead bile from the liver and the gallbladder, through the pancreas, and into the upper portion of the small intestine. Bile is a liver-produced fluid that helps the body digest fats. If the biliary ducts are clogged, the passage of the bile through to the intestines is hindered. Symptoms can include burping, nausea, vomiting, a pain in the back, low pressure, cramps in the stomach, and an attack of the gallbladder. All the diseases, which are caused by clogged bile ducts, are dangerous for human health, as the liver and pancreas are key organs without which one cannot live. In addition, in the case of partial damage, the quality of life is significantly reduced. Risks and complications of obstructed bile ducts are jaundice and liver disease, acute and chronic inflammation of the pancreas, inflammation of the gallbladder and the bile ducts, gallbladder perforation, and perforation of the bile ducts. In addition, obstruction jaundice can cause a blood clotting disorder, decreased hepatic function, and cholangitis. Cholangitis can have a bad influence on the cardiovascular system and kidneys [99,100,101,102,103,104]. Biliary obstruction is classified into five types: (1) an obstruction is located further than or equal to 2 cm from the main hepatic confluence, (2) an obstruction is located less than 2 cm from the main hepatic confluence, (3) the ceiling of the biliary confluence is intact, right ductal system communicates, (4) the ceiling of the biliary confluence is intact, left ductal system communicates, and (5) the ceiling of the confluence is destroyed, while bile ducts are separated (see Figure 5) [105].

When the strictures are close to hepatic confluence, treatment is more difficult. Treatments of biliary obstruction are surgery or endoprosthesis—stents. Treatment with stents is preferred over surgery techniques because it avoids the risk of liver puncture, and it is an easier process [106].

### 4.2. Types of Biliary Stents

**Figure 6:**
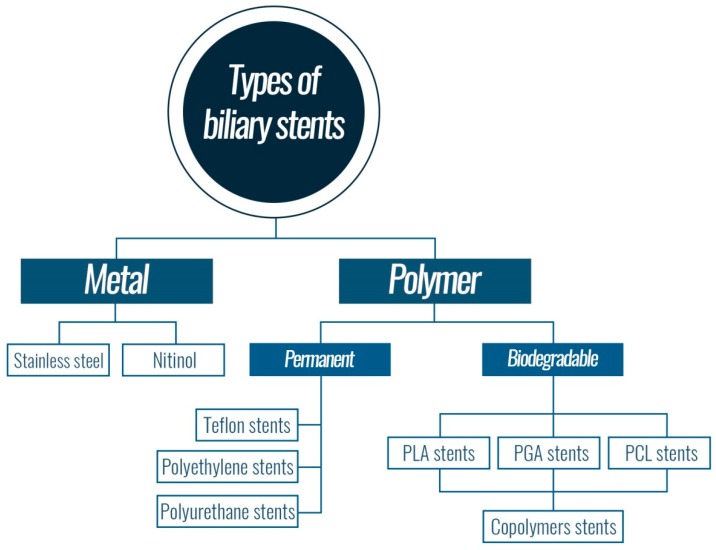
Types of biliary stents.

Currently, biliary stents made on the basis of polymers or metals are used (Figure 6). Polymer stents have controllable properties and are cost effective [107]. However, the use of polymeric stents is limited by clogging, and they are only applicable for a short time. Self-supporting metal stents are preferred for long-term use. Their disadvantage is that they are more expensive than polymer stents. Both polymer and metal stents have a tendency to occlude with time and then must be operatively removed. The innovative solution is a biodegradable polymer stent that disintegrates gradually [108].

#### 4.2.1. Metal Stents

Self-expandable metal stents are widely recognized as an option for the palliative treatment of patients with malignant biliary obstructions and with an expected survival of more than four months [109]. These stents are made of either stainless steel or nitinol [110]. Metal stents are usually preloaded in a covered polymer sheath. Stents with a diameter of around 2.7–2.8 mm are the most frequently used [111]. When a stent is fully expanded, its diameter can reach 10 mm [112] and has a significantly longer patency rate compared to polymer stents [113]. The biggest disadvantage of metal stents is its cell ingrowth into the stent and emergence of restenosis and its high price [114].

#### 4.2.2. Permanent Polymer Stents

The first use of polymer stents in 1980 was for a patient with a malignant bile duct obstruction [115]. Polymer stents are divided into two types: (i) permanent stents and (ii) biodegradable stents. They are produced in many sizes, shapes, and lengths (3–15 cm). The most used stents are straight and pigtail types. These stents are made of polyethylene or Teflon [116]. Most models of polymer stents are curved to fit the contour of the common bile duct and to prevent stent migration. The first stents were produced with side holes, but these side holes caused the creation of sludge. Permanent biliary polymer stents for temporary use contain side flaps that are intended to prevent stent migration. These are known as “Tannenbaum” stents and have double layers. A second layer was added to prevent a sludge formation [117]. Sludge is a mixture of bile, blood, and occasionally bacteria that adhere to the surface of the stent. The main problem of polymer stent use is their tendency to occlude with time, which leads to jaundice and cholangitis. The inner surface of the stent is occluded by a biofilm containing components of bacteria and bile [118]. The special design of Tannenbaum stents has been used to prolong the patency. However, further randomized controlled studies did not support these results [119,120,121]. Other studies have showed that the most effective method to prolong stent patency is by inserting a stent of a larger diameter (3–3.8 mm) that remains unclogged for a significantly longer period when compared to smaller stents (2.3–2.8 mm) [122,123]. It can be concluded that stent diameter has a considerable effect on stent patency. The larger the stent, the longer the patency as well as the functionality of the stent.

##### Polyethylene and Polyurethane Stents

Polyethylene (PE) and polyurethane (PU) stents are an alternative to metal stents, and they are much cheaper than metal stents. The first permanent polymeric stent was introduced in the 1980s [124]. These polymers are commonly used material. However, PE and PU are not the best choice because they are easily clogged with bile, they migrate, and they have to be removed surgically [125].

##### Teflon Stents

The majority of polymer stents are made of PE, PU, or Teflon. Stents made of these materials are permanent. Their main complication is clogging, leading to the need for a timely replacement of the stent every three to six months. Previous studies suggest that Teflon stents are better than stents made of PE with regard to their resistance to occlusion. In addition, Teflon has a significantly lower coefficient of friction compared to other polymers. This smoothness may impede the adhesion of bacteria and body fluids. In one study [84], the authors focused on Teflon stent testing, in which two types of stents with different diameters were compared. One stent was covered with antireflux sleeve and the other stent was without an antireflux sleeve. The antireflux sleeve was intended to prevent the backflow of the duodenal contents into the bile system. However, the achieved results were not satisfying because, while at the beginning the patency of the stent with the antireflux sleeve was obtained, in the next phase, the clogging occurred [126].

##### Biodegradable Stents

**Figure 7:**
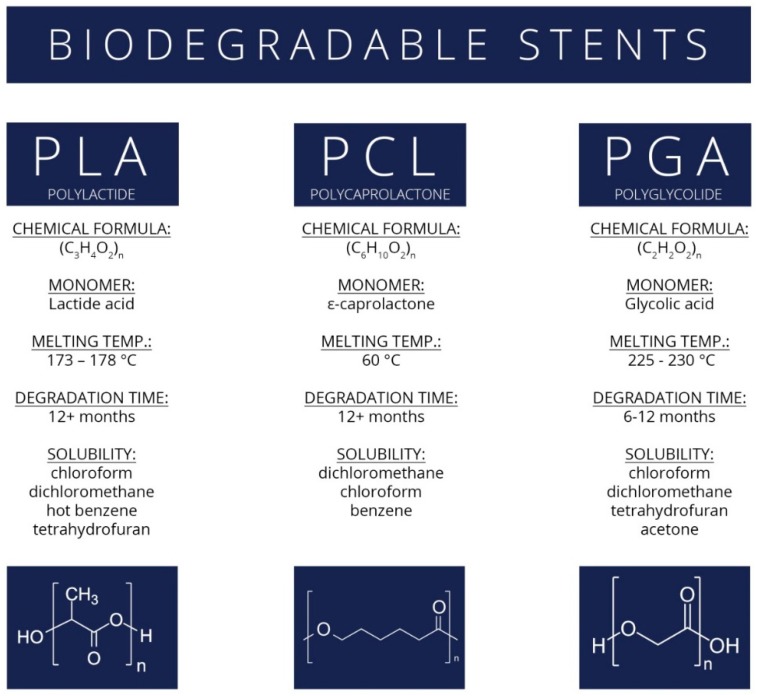
The main biodegradable polymers for biliary stents and some physicochemical characteristics.

Biodegradable stents are an attractive alternative to self-expanding stents in the treatment of biliary, coronary, and other duct diseases. These stents can be made of biodegradable polymers [127,128,129]. Biodegradable polymers, such as polycaprolactone (PCL), poly(L-lactic acid) (PLA), poly(glycolide) (PGA) (properties of the named polymers are summarized in Figure 7), and their copolymer poly(L-lactide-*co*-glycolide) (PLGA), have attracted much attention due to their excellent mechanical properties. These polymers can be used in biomedical applications, such as surgical sutures, drug carriers, tissue—engineering scaffolds, implants for interior bone fixation, and other temporary medical devices [130,131,132,133,134]. The polymers break down into basic components that are not toxic for the body. Biodegradability time varies depending on the polymer composition.

##### Polylactide Stents for Biliary Duct

**Figure 8:**
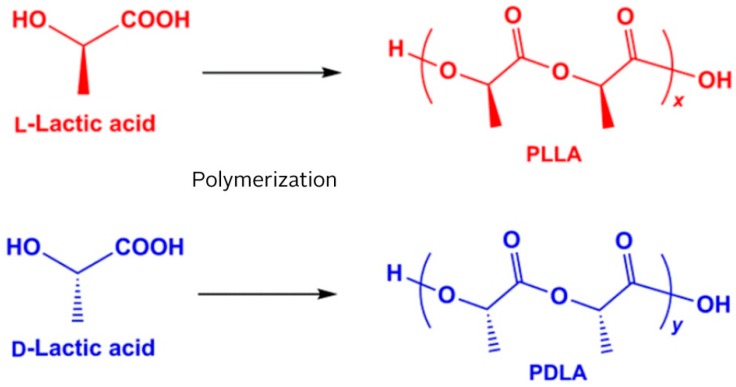
Poly(L-lactic acid) (PLLA) and poly(D-lactic acid) (PDLA) forms of polylactic acid (PLA).

Biliary stents based on polylactide were examined, such as alternatives to metal and polymer stents. PLA is a polymer made of lactic acid, which has two optical isomers L- and D-. The resulting PLA can be present in one of three forms: PLLA, PDLLA, and PDLA. The stereochemistry and ratio of L- and D-isomers influence PLA properties (Figure 8) and biodegradability. PLA with an L-isomer content (PLLA) greater than 90% tends to be crystalline, while one with lower optical purity is amorphous [135].

**Figure 9:**
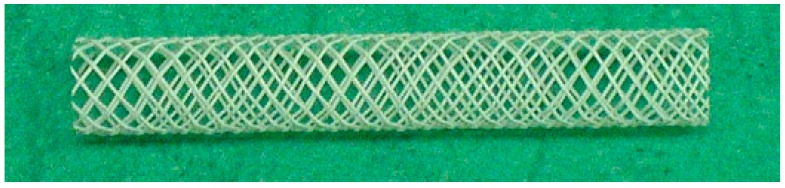
Self-expanding PLA BaSO_4_ stent (fully expanded), reproduced from [140], with permission from Elsevier, 2019.

PLA has good mechanical properties, such as transparency, high strength, and biodegradability [136,137,138]. The mechanical properties of PLA may vary depending on various parameters such as crystallinity, molecular weight, material formulation (plasticizers, blends, composites, etc.) and processing. The resulting polymer may range from soft and elastic materials to stiff and high-strength materials [139]. Thus, other chemical constituents are normally added into PLA to adjust the properties of biliary stents. In one study [140], fibers were produced by melt spinning a blend of PLLA and BaSO_4_. Fibers were braided to form a tubular mesh, and the mesh was hot-threaded to stabilize the structure of the stent. The stent wall thickness was 0. 25 mm, the length was 50 mm, and the outer diameter was from 6 to 7 mm when fully expanded (Figure 9). The stents were consequently inserted into commercial applicators for stents and applied to six pigs (group A) which had a disease of the bile duct. After being applied, the stents opened almost immediately to their full diameter. Stents of polyethylene were inserted into another six pigs (group B) to compare the efficiency.

The pigs were observed for six months, and the volume of the bile output in the drain sac was measured daily. In group A (with biodegradable stents), the total output of bile was significantly smaller compared with group B (with polyethylene stents). After six months, it was summarized that, while the accumulation of bile in group A was about 165 mL (median), in group B, it was about 710 mL (median). In both groups, the stents were seen to be in place after three months. After six months, in group A, the stents were not detected. The study found that the stent under investigation is safe and effective in the treatment of postcholecystectomy cystic-duct leakage [140].

PLA-BaSO_4_ biliary stents were also used in another study [141]. They investigated the method of hepaticojejunal anastomosis (HJ) due to the fact that the application of the conventional method is very difficult in a non-dilated bile duct with a median diameter less than 4 mm. They used the PLA-BaSO_4_ biodegradable biliary stent for the conventional HJ technique performed in a non-dilated white channel size of 7–9 mm [140,142]. The study showed good results achieved with this method. Advantages can be summarized as follows: It was not more difficult than the conventional surgical method, it did not appear to increase the number of complications, and it did not require any subsequent stent removal and used biodegradable materials. The biodegradable stent method can provide similar healing to a conventional method, but using fewer stitches and easier techniques [140].

Another study [143] was aimed at the toxicity of PLA in combination with BaSO_4_. The chemicals were used in a proportion 96:4 (PLA:BaSO_4_). Fibers of 0.3 mm in diameter and 5 mm long were prepared from this material and used for stent manufacture. This stent made of fibres was applied to male rats. The rats were anesthetized after one, three, seven, and twenty-one days after the applications, and histological and pathological tests were carried out. No histological changes were observed. The study found that the material used was no more toxic than the reference steel material [143].

**Figure 10:**
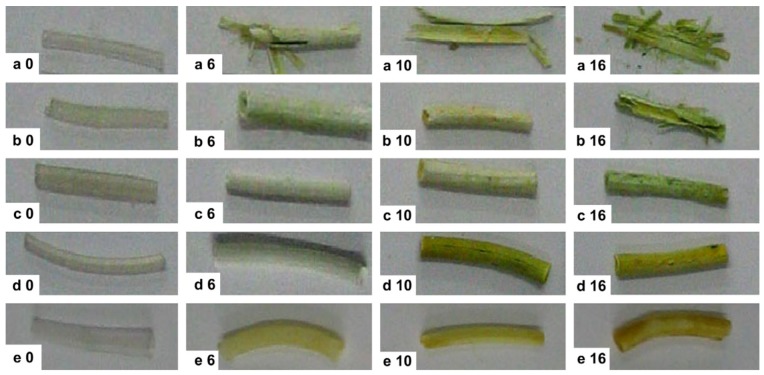
Optical images of PLGA stents in different degradation stages (days) in bile at 37 °C in vitro. Lactic acid/glycolic acid (LA/GA) (**a**) of 50/50; (**b**) 60/40; (**c**) 71/29; (**d**) 80/20; and (**e**) 88/12, reproduced from [144] with permission from Elsevier, 2019.

In another study [144], the degradation behavior of biodegradable polymeric stents based on poly(L-lactide-*co*-glycolide) (PLGA) in the bile duct was evaluated. The PLGA samples were synthesized in various molar ratios of lactide (LA) and glycolide (GA) (ratios of LA/GA: 88/12, 80/20, 71/29, 60/40, 50/50). Polymers were processed into circular tubing with parameters of 40 mm length, 10 mm outer diameter, and a wall thickness of about 2 mm. The stents were placed into human bile to determine the degradation behavior in vitro. Changes in composition, mass loss, molecular weight configuration, morphology, and water uptake were observed. Copolymers were prepared by ring opening copolymerization of LA and GA with a catalyst (stannous octoate—Sn(Oct)_2_, 95%) and used for stent preparation with a length of 40 mm, an outer diameter of 10 mm, and an inner diameter of 6 mm. Stents with various molar ratios were placed in glass bottles filled with 20 mL bile of clinical patients. The samples were incubated at 37 °C under the oscillation of bile for twenty days. The bile was replaced every day, and the samples were taken out from the bile every two days. The samples were cleaned using distilled water and dried under vacuum at room temperature. The PLGA stents (LA/GA = 71/29) (Figure 10) displayed a suitable duration and degradation behavior for in vivo tests. Thus, they were sterilized and implanted in adult male Wistar rats. Five rats were sacrificed every week to examine the degradation state of the stents within a nine-week period. 

In vitro experiments revealed a change in the color of the stents from originally translucent to yellowish. The color change was due to water and bile absorption. After several days, the stents were slightly expanded, but the functions of the stents were maintained. Later, stents were deformed and cracked, depending on the copolymer composition (PLGA 50/50 after six days, PLGA 60/40 after sixteen days, PLGA 71/29 after twenty days). Another two samples preserved the shape, but they were more stiff and brittle after twenty days. Tests of PLGA stents on animals were carried out based on in vitro tests (71/29). It was observed that the stents were expanded in two weeks. They became deformed at three weeks, but the functions of the stents were maintained. The stents disappeared completely after five weeks of surgery. The study has shown that a biodegradable stent made of PLA and PGA is suitable for short-term use [144].

In another study [145], self-expanding biodegradable stents were used in a porcine model of biliary stenosis. The stents were prepared from a copolymer consisted of L-lactide and D-lactide (96:4). D-lactide was used for polymer crystallinity decrease and to accelerate the absorption rate. Stents were applied to pigs for six months, and the study concluded that no clinical evidence of biliary obstruction appeared in this study. The study found that the bio absorbable biliary stent is suitable for endoscopic application and for approximately six months [145].

In one study [146], an application of a biodegradable polymer stent for the repair and reconstruction of the bile duct was investigated. PLGA in ratio LA/GA 80/20 was used for copolymer preparation. Copolymer was treated in a circular tube and studied to determine in vitro degradation behavior in the bile. Copolymer tubes were further used for tests in dogs′ biliary systems. The obtained results showed that the PLGA stents exhibited the required biomedical properties. They spontaneously disappeared from bile duct after four or five weeks. The biodegradation time was sufficiently long for the repair and reconstruction of the bile duct [146].

In another study [147], helical PLLA stents were used for in vivo and in vitro tests. For in vivo tests, canine models with injuries of bile duct were used, and a transection of the common bile duct was performed. Duct to duct anastomosis was performed using helical PLLA biodegradable stents. Characterization of PLLA stents was performed after three months. PE and PLLA membranes were inserted into human bile to follow in vitro study. After two months, samples were withdrawn and observed using SEM. The results showed that biodegradable stents had a good self-clearing effect to clear away the attached sludge as well as sufficient biocompatibility. The biodegradation provides the stent self-clearing effect which prevents an attachment of bile sludge. The self-clearing property could extend applicability of the stent in the bile duct [147].

##### Polycaprolactone, Polyglycolide, and Polydioxanone Stents

PCL is a well-known polymer that is attractive to the biomedical research community due to its structural stability, biocompatibility, long biodegradability time, and capacity to form blends with a many other polymers. One of the first PCL materials was synthesized in the 1930s [148], and first studies were focused on drug delivery and surgical threads. In the early 1990s, interest in PCL began to grow again, by the development of tissue engineering, which deals with the regeneration, repair and building of new tissues or organs, by combining knowledge about cells from biology and medicine [149,150,151]. Nowadays, there are many PCLs with very different properties for many medical applications [152]. PCL is combined with other polymers to form copolymers, which have better properties than PCL itself. Other biodegradable polymers are used for copolymer preparation, polymers such as polydioxanone (PDO), polylactide (PLA) or polygycolide (PGA). Combinations of these polymers bring better properties to the resultant material, such as biodegradability or healing promotion.

In the study [153], a copolymer of PCL and PLA reinforced with PGA fibres was used for regenerating the tube of a bile duct. A bioabsorbable copolymer was seeded with autologous bone marrow cells which had been removed from the swine sternum. After one hour, stents were implanted into the pigs from which bone marrow cells had been taken. Six months after implantation, all pigs were alive and gaining weight without signs of jaundice or icterus. They were sacrificed, and histological and macroscopical observation of the bile duct showed that the neo-bile duct was practically similar to the native common bile duct in morphology. The results showed that the polymeric stent was replaced by an extra hepatic bile duct, carrying bile to the duodenum without any leakage in the peritoneal cavity while preserved its tubular form in the short-term following implantation [153].

Braided stents of PDO with a length of 30 mm and diameters of 6, 8, and 10 mm were used in another study [154]. Stents were placed into the common pigs’ bile ducts and the animals were clinically monitored daily. The pigs were gradually sacrificed two, eight, thirteen, and twenty weeks after implantation, and the bile ducts were observed. Neither bile duct obstruction nor postsurgical complications were observed. The study found that biodegradable PDO-based polymer stents are suitable for biliary applications with a treatment period of about 13 weeks [154,155]. 

## 5. Challenges for New Materials with Antimicrobial Properties

Infectious diseases are always caused by some kind of microorganism. There are several ways how the microorganisms can enter a human body, one of them being a biological contamination of the surface. Biomedical devices and implants are no exception [156,157]. Microbial organisms can adhere to the implanted surface or to the surface of a biomedical device and produce bacterial biofilms [77]. A growing colony of microbial organisms causes chronic infections that are very resistant to antibiotics [156,157]. The solution to this complication is the production of biomedical instruments and implants that will have lethal antimicrobial properties [158,159,160].

Antimicrobial (bio)materials are defined as materials that have antimicrobial properties and a resistance to infection. These materials are also able to be a carrier of species whose main task is to prevent, treat, or inhibit potential infection [161]. Technological inventions in the nanotechnology field are continually provided by advanced antimicrobial materials used for medical applications. At present, intensive efforts are centered on the development of innovative antimicrobial materials that are made by the physical or chemical modification of surfaces and matrices. This process can develop materials that are convenient for applications in the medical field and at the same time have an antimicrobial effect [162].

Antimicrobial activity of a material can be obtained by two different strategies. The first strategy is to release a chemical or antimicrobial substance from the surface of a material that focuses on bacteria around the material. The second strategy is antibiotic molecules that do not allow the attachment of bacteria being grafted onto the surface of the material [163]. Antimicrobial properties can be supplied by different agents such as silver, gold, copper, zinc, or clay materials and their modifications [164]. Metallic nanoparticles, especially noble metals, are more attractive to scientists because of their unique electrical, catalytic, and optical properties [165,166,167,168]. They have a high surface-to-volume ratio and strong antibacterial and antimicrobial properties.

The antimicrobial agents are used to modify the biodegradable polymeric materials from which they are slowly released and prevent the formation of a biofilm on the surface of the polymeric material. The antimicrobial effect can also be given to the material by a physical surface treatment where bacteria and other microorganisms are unable to adhere and develop into a biofilm. Other options based on the physical properties of the surface, such as a roughening or smooth surface, super hydrophobia, etc., have also been proposed to prevent bacterial adhesion [169,170].

The research of new antimicrobial agents is important as microorganisms are increasingly resistant to antibiotics. At present, antibiotic resistance is considered to be one of the biggest health challenges according to the World Health Organization (WHO) [165,168]. The first confirmation of bacterial resistance is dated to 1967, when penicillin-resistant *Streptococcus pneumonia* was described in Australia. At present, resistance is not limited to a particular strain of bacteria or to a specific antibiotic. The Centers for Disease Control and Prevention (CDC) noted that more than 70% of all bacterial infections resist at least one of the major antimicrobial substances commonly used in hospitals [166]. Moreover, antibiotic resistance is not the only problem for any particular country. Global monitoring data collected by the WHO have found that approximately 79% of bacteria developed resistance to one or more antibiotics [167].

### 5.1. Antimicrobial Agents

#### 5.1.1. Silver

Most attention is drawn to silver nanoparticles, which are currently used in pharmaceutical products, such as ointments and creams, but also bandages that prevent infection from burns and open wounds [171,172,173,174,175,176]. Silver has long been used as an antimicrobial agent that can be used in various applications [177,178,179,180,181,182,183,184,185] and which, at a certain concentration, does not show toxicity to the human organism. Due to the strong antimicrobial nature of silver cations, they are used, for example, for the production of vascular, urinary, and peritoneal catheters, vascular grafts, surgical sutures, or implants for fracture fixation [186]. Silver ions have been shown to counteract many species of pathogens that may occur during implantation, such as the bacterial strains *E. coli*, *P. aeruginosa*, *S. epidermidis*, *S. aureus*, etc. Silver ions are effective against these bacteria because Ag^+^ ions disrupt the function of the bacterial cell membrane, metabolic proteins, and enzymes by linking to DNA and thiol groups in proteins [187,188]. Silver ions accumulate on the surface of the cell membrane of the bacteria, subsequently altering the permeability of the bacterial membrane, causing considerable membrane damage [189]. They are used in various forms, such as particles, silver salts, chelates, or fillers in a matrix [190]. 

In medical applications, silver nanoparticles (NP) are most commonly used, which exhibit high bactericidal activity at concentrations that are not toxic to human cells, and also significantly increase the antimicrobial activity of conventional antibiotics. The physicochemical properties of silver NPs facilitate interaction with the bacterial membrane. Particle size, shape, and surface play a very important role [191,192]. One of the most important aspects to determine silver NP interaction with cells is their size. The smaller the NPs, the larger the ratio of the surface area is to the nanoparticle volume. This makes it possible for them to interact more with cell membranes than with larger particle sizes. The most effective NPs of silver include particles in a range of 1–10 nm, which show the highest antimicrobial activity and simultaneously the fastest interaction with the cell membrane [189,193].

Currently, scientists are dealing with the bacterial resistance to silver. Bacteria exposed to sub-inhibitory concentrations of silver NP have been found to be able to form a resistance to antibiotic activity. This is caused by a production of flagellin, a bacterial adhesion protein, that causes the aggregation of silver NPs to eliminate their antimicrobial effect against Gram-negative bacteria [188].

#### 5.1.2. Copper

Copper ions and their alloys (brass, bronze, copper-nickel, etc.) are also studied for their antimicrobial properties in a wide range of microorganisms and rapid antimicrobial activity. The antibacterial and antimicrobial properties of copper are currently used in water purification, paints, building materials, and the textile industry. Copper has been found to act primarily against Gram-positive bacteria, such as *S. aureus*, but is also active against Gram-negative bacteria such as *E. coli* [194,195,196,197]. Excess of copper accumulates in the body causing severe metabolic dysfunction and contributes to the development of neurodegenerative diseases. This raises concerns about whether the amount of doped copper might be safe for medicine uses [198].

#### 5.1.3. Zinc Oxide

Recently, metal oxides were studied for applications as antimicrobial agents. Metal oxides are inorganic compounds that have strong antimicrobial activity at low concentrations [199]. They are stable, non-toxic, and may bring mineral elements that are important for the human body [200,201]. Among metal oxides, ZnO demonstrates a significant antimicrobial effect. It inhibits the growth of a wide spectrum of bacteria [202]. This metal oxide disrupts the integrity of the bacterial membrane and other cell processes. Particles with a large surface area have stronger antimicrobial activity than particles with small surfaces [199,203]. Particularly, ZnO NPs with sizes less than 100 nm have strong antimicrobial activity, better than micro- or macro-sized particles. Researchers have shown that ZnO NPs have a selective toxicity to bacteria, but exhibit a minimal effect on human cells [199,204,205].

#### 5.1.4. Clay Minerals

**Figure 11:**
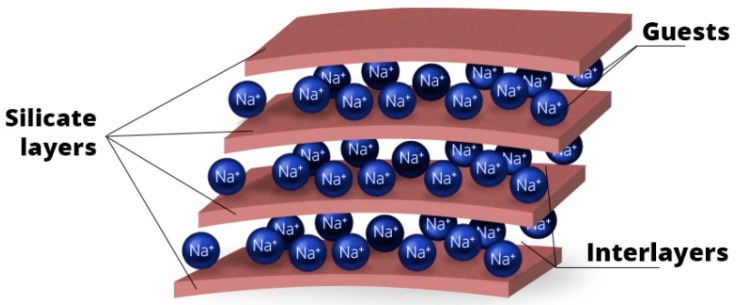
Layered structure of clays.

Metal ions are good antimicrobial agents, but their application is limited because of potential environmental problems that could be overwhelmed by toxic metals in the future. For this reason, other possible materials with low manufacturing costs, high microbial activity, and long service life are studied [206]. Clay minerals attract attention due to their non-toxicity to the environment and easy modification by intercalation [207]. They are very interesting materials among others due to their layered structure in which various organic or inorganic compounds with antimicrobial and antibacterial properties can be incorporated. The layered structure (Figure 11) consists of silicate layers and interlayer spaces into which another compound or guest may be incorporated (intercalation). The clay minerals such as vermiculite or montmorillonite have a naturally layered structure, and they are suitable materials for intercalation of wide range of chemicals.

Intercalation of organic compounds into layered inorganic clay minerals provides an easy way to prepare organo-inorganic hybrids that have both an inorganic host and organic guest properties [208]. A variety of antimicrobial agents such as metal ions, hexadecyltrimethylammonium bromide (HDTMA), hexadecylpyridinium bromide (HDP), or chlorhexidine (CA) have been used as fillers. They seem to have a great potential for preparation of biodegradable polymeric materials with antimicrobial properties.

#### 5.1.5. Hydroxyapatite

Natural materials are often researched for medical applications, as they are usually readily available and environmentally friendly. An example is hydroxyapatite (HA). HA is a naturally occurring mineral form of calcium apatite, which also occurs in hard human tissues such as bones and teeth. Currently, HA is broadly used in medicine as modern body implants (teeth, hip joints, bones). HA is a suitable material for biomedical applications, since it is itself antimicrobial, highly compatible with the human organism, and supports the growth of the original bone tissue [209]. A disadvantage of using of hydroxyapatite for the preparation of polymer composites is the mechanically unsuitable properties of the resulting material, such as poor strength or inability to biodegradate. 

#### 5.1.6. Carbon Nanomaterials

Carbon-based nanomaterials are widely studied for their excellent physicochemical, mechanical and optical properties. These materials have also been studied in recent years for their antimicrobial effects [210,211]. Carbon based nanomaterials such as graphene, graphene oxide, reduced graphene oxide, carbon nanotubes, and others have a specific structure that has partial antimicrobial effects. Graphene based nanomaterials may also be an excellent carrier of other antimicrobial agents [212,213,214,215,216,217]. The one with the greatest potential is graphene oxide.

##### Graphene Oxide with Antimicrobial Agents

Graphene oxide (GO) is a fascinating carbon nanomaterial. Specifically, graphene oxide is an oxidized derivative of graphene. GO contains a large number of oxygen-containing functional groups such as hydroxyl and epoxy functional groups localized on the basal carbon lattice and carboxyl groups at the edges. These functional groups are active sites for functionalization and hybridization using other materials such as metal ions and metal oxides [218].

Some reports describe the very strong antimicrobial activity of graphene oxide (GO) [213,219]. Some studies have reported that carbon nanomaterials are cytotoxic for bacteria, but cytotoxicity is dependent on the amount of carbon nanomaterials and their size [213,220]. However, many studies show the poor results of antibacterial tests; some research has even showed GO supporting the growth of bacterial colonies [221]. Graphene oxides have a good biocompatibility. For this reason, it is a suitable material for antibacterial agent modification.

ZnO/GO composites were prepared by Wang et al. [218], and antimicrobial activity was tested. The *E. coli* strain and human HeLa cells were used to study antibacterial activity and cytotoxicity. The composite showed strong antibacterial activity at low concentrations, which did not threaten HeLa cells [218].

In another study [222], the antibacterial activity tests of ZnO/graphene composite showed a 100% inhibition of *E. coli* in the medium after twelve hours. Antibacterial activity was probably caused by the physical interactions of graphene with bacterial membranes, which were disturbed. The antibacterial properties of ZnO may be due to their ability to photocatalytically generate H_2_O_2_ and also to penetrate cells and disrupt the bacterial membrane upon contact with ZnO NPs. Both of these compounds synergically cooperated and resulted in the significant antibacterial activity [222].

Graphene oxide is a good material for the modification with silver NPs. Connecting the physical properties of GO and the strong antibacterial activity of Ag NPs may create a very strong antibacterial material. This assumption was verified in some studies [223,224,225] in which composites of GO and silver NPs (GO–Ag) were produced and characterized. The researchers showed excellent antibacterial activity against the Gram-negative bacterial strain *P. aeruginosa*. The composite was tested determining a minimum inhibitory concentration (MIC), and the results showed an antibacterial activity of GO–Ag nanocomposite between 2.5 and 5.0 µg/mL. Another result confirmed 100% inhibition of *P. aeruginosa* cells after one hour [223,224,225].

##### Carbon Nanotubes

Carbon nanotubes (CNTs) are formed by graphenic layers containing carbon atoms in sp^2^-hybridized state, which are rolled up into hollow, cylindrical arrangement. CNTs were tested in many studies as carriers of antimicrobial agents such as metal ions or organic compounds. CNTs enriched with superparamagnetic properties due to iron oxide and antimicrobial due to silver nanoparticles have been investigated for their antimicrobial properties and for magnetic separation [226]. The nanotubes were tested to treat an aqueous medium containing *E. coli*. The antimicrobial activity was confirmed. Superparamagnetic properties were used to prevent the release of silver NP while maintaining antimicrobial properties [226].

#### 5.1.7. Antimicrobial Polymeric Materials

Various processes are currently used to form antimicrobial polymeric materials. As an example, the surface functionalization or modification of the filler can be mentioned. Cleavage of functionalized polymers on the surface of the material can cause antibacterial activity. Some studies deal with the derivatives of antimicrobial polyhexamethylene guanidine (PHMG) (the salt of stearic acid, sulphanilic acid salt, and PE wax) that were added to biodegradable materials. PHMG affects the electrical properties of the surface of the polymer, which subsequently becomes antibacterial [227]. Similarly, chitosan can be used, which belongs to a group of polycationic polymers that provide bactericidal activity by interrupting the negative charge of the bacterium membrane which subsequently goes to the death [228].

When the biodegradable polymer is modified with antibacterial filler, such as silver or modified clay material, by progressively degrading the polymer, the antibacterial component is released and subsequently causes the death of the microorganisms. The polymer product should perform its function throughout the treatment, while at the same time it should gradually degrade and release the antibacterial agent [229].

Antimicrobial peptides have evolved as part of the immune system of a variety of organisms, including humans, and, as their name suggests, are able to kill pathogenic microorganisms such as bacteria, yeasts, or viruses quickly and effectively [230,231]. Antimicrobial peptides act using a mechanism that allows them to act only on pathogenic microorganisms while not harming the cells of the human body. This selectivity is due to the differences in the cell structure. Bacterial cells have membranes composed of phospholipids bearing a negative charge (phosphatidylglycerol, phosphatidylserine, and cardiolipin). These, together with other surface structures (the cell wall in Gram-negative or lipopolysaccharide in Gram-negative bacteria), give the cells an overall negative charge, thereby electrostatically attracting cationic antimicrobial peptides. In contrast, human (animal) cells contain rather neutral phospholipids (phosphatidylcholine and phosphatidylethanolamine) and cholesterol in their membranes, which additionally stabilizes the membrane. Antimicrobial peptides interact with the bacterial membrane, immerse in it with their hydrophobic portion and form pores (or otherwise interfere with its integrity), leading to the release of vital substances from the cell and its death. There is, however, also a group of antimicrobial peptides that do not break the membrane, only pass through it inside the cell, and block some of the metabolic processes. Importantly, antimicrobial peptides act rapidly, on the order of tens of minutes, and rather by a physical mechanism across the cell surface, so microbes are unlikely to develop resistance to them [232,233,234,235,236].

## 6. Conclusions

Stenting is a breakthrough medical method for restoring body fluid flow in various body ducts. One of the main problems with their use is the need to remove them from the body for a variety of reasons, such as clogging due to biofilm formation, infection due to the presence of a biofilm, but also due to migration in the body or termination of the treatment process. Removal is performed by surgical intervention, which can endanger a patient’s life. The solution to these problems may be biodegradable polymeric materials that progressively disintegrate into basic components that the body can break down. Simultaneously, biodegradable polymers can be easily modified with antimicrobial agents, drugs, or antibiotics to prevent the formation and growth of a biofilm on their surface and the development of infectious diseases. The antimicrobial agents are slowly released when the stent is degraded and prevent bacteria and microorganisms from adhering to the stent surface, thereby avoiding biofilm formation and subsequent stent clogging. There are many antimicrobial agents with a well described effect. However, their usage is complicated by many factors, such as specific effects only for selected bacteria and other microorganisms, the limited period of antimicrobial agent release, the possible toxic effect on the human body, or the environment. The preparation of new efficient materials which can be based on biodegradable polymers containing one antimicrobial agent or their combination with a wide range of antimicrobial effects is still a challenge.

## Figures and Tables

**Table 1:** Host products affecting bacterial attachment to a biomaterial [74].

Environment of the Biomaterial	Suspended Liquids	Main Components of the Host Products
Urethra	Urine, prostatic fluid	Mucopolysaccharides, Tamm Horsfall proteins, glycoproteins
Cardiovascular	Blood	Serum, albumin, fibrinogen, fibronectin
Ocular	Tears	Proteins (lysozymes), fibrin
The oral cavity	Sputum	Mucopolysaccharides, glycoproteins, serum albumin
Intestinal tract	Digested food and liquids	Mucopolysaccharides, glycoproteins, serum albumin, steroids
Biliary ducts	Bile	Steroids, bile salts, mucopolysaccharides
Respiratory passageways	Respiratory secretions	Mucopolysaccharides
Bones, joints	Synovial fluids, blood	Serum proteins, blood elements

**Table 2:** Examples of implant infections.

Implant	Organisms	Associated Disease	References
Prosthetic valve	*Staphylococcus aureus, Staphylococcus albus*	Prosthetic valve endocarditis	[80]
Contact lenses	*Pseudomonas aeruginosa, Staphylococcus epidermidis*	Keratitis	[75]
Intravascular catheters	*Staphylococcus epidermidis, Staphylococcus aureus*	Septicaemia, endocarditis	[81]
Urinary catheters	*Escherichia coli, Pseudomonas aeruginosa, Enterococcus faecalis*	Bacteraemia	[82]
Joint replacement	*Pseudomonas aeruginosa, Staphylococcus epidermidis, Staphylococcus aureus*	Septicaemia, device failure	[83]
Endotracheal tube	*Pseudomonas aeruginosa, Escherichia coli, Ureaplasma urealyticum, Staphylococcus aureus*	Pneumonia	[84]
Biliary stents	*Enterobacteriaceae*, *Pseudomonas aeruginosa, Enterococcus, Candida albicans*	Biliary obstruction, septicaemia	[85]
